# Hydriodate of Potash

**Published:** 1826-07

**Authors:** 


3. *Hydriodate of Potash.* M. Lisfranc has given the particulars of a remarkable case of scrofulous enlargement of the female mamma, which resisted all the usual means of resolution, but ultimately gave way to frictions with the iodine ointment.

